# The effect of order–disorder phase transitions and band gap evolution on the thermoelectric properties of AgCuS nanocrystals†
†Electronic supplementary information (ESI) available: Powder XRD pattern of AgCuS nanocrystals obtained after 12 h reaction (Fig. S1), XPS spectra of AgCuS nanocrystals (Fig. S2), FESEM image of AgCuS nanocrystals after sintering (Fig. S3), zoomed Raman spectra (Fig. S4), electrical conductivity (*σ*) of bulk and nanocrystalline AgCuS (Fig. S5), two cycle heating cooling Seebeck coefficient data (Fig. S6), temperature dependent thermal conductivity (*κ*_total_) of nanocrystalline AgCuS, power factor (*σS*^2^) and thermoelectric figure of merit (*ZT*) of bulk and nanocrystalline AgCuS (Fig. S7) and schematic representation of band gap evolution during phase transition (Fig. S8). See DOI: 10.1039/c5sc02966j


**DOI:** 10.1039/c5sc02966j

**Published:** 2015-10-08

**Authors:** Satya N. Guin, Dirtha Sanyal, Kanishka Biswas

**Affiliations:** a New Chemistry Unit , Jawaharlal Nehru Centre for Advanced Scientific Research (JNCASR) , Jakkur P.O. , Bangalore 560064 , India . Email: kanishka@jncasr.ac.in; b Variable Energy Cyclotron Centre , 1/AF Bidhannagar , Kolkata 700064 , India

## Abstract

Copper and silver based chalcogenides, chalco-halides, and halides form a unique class of semiconductors, as they display mixed ionic and electronic conduction in their superionic phase. These compounds are composed of softly coupled cationic and anionic substructures, and undergo a transition to a superionic phase displaying changes in the substructure of their mobile ions with varying temperature. Here, we demonstrate a facile, ambient and capping agent free solution based synthesis of AgCuS nanocrystals and their temperature dependent (300–550 K) thermoelectric properties. AgCuS is known to show fascinating p–n–p type conduction switching in its bulk polycrystalline form. Temperature dependent synchrotron powder X-ray diffraction, heat capacity and Raman spectroscopy measurements indicate the observation of two superionic phase transitions, from a room temperature ordered orthorhombic (β) to a partially disordered hexagonal (α) phase at ∼365 K and from the hexagonal (α) to a fully disordered cubic (δ) phase at ∼439 K, in nanocrystalline AgCuS. The size reduction to the nanoscale resulted in a large variation in the thermoelectric properties compared to its bulk counterpart. Temperature dependent Seebeck coefficient measurements indicate that the nanocrystalline AgCuS does not display the p–n–p type conduction switching property like its bulk form, but remains p-type throughout the measured temperature range due to the presence of excess localized Ag vacancies. Nanocrystalline AgCuS exhibits a wider electronic band gap (∼1.2 eV) compared to that of the bulk AgCuS (∼0.9 eV), which is not sufficient to close the band gap to form a semimetallic intermediate state during the orthorhombic to hexagonal superionic phase transition, thus AgCuS nanocrystals do not show conduction type switching properties like their bulk counterpart. The present study demonstrates that ambient solution phase synthesis and size reduction to the nanoscale can tailor the order–disorder phase transition, the band gap and the electronic conduction properties in superionic compounds, which will not only enrich solid-state inorganic chemistry but also open a new avenue to design multifunctional materials.

## Abstract 2

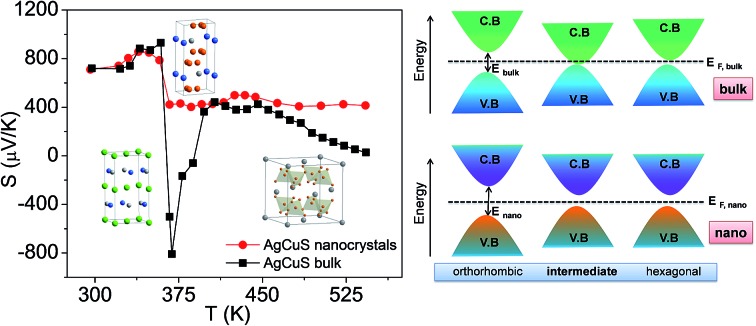
The present study demonstrates an ambient solution phase capping free synthesis of superionic AgCuS nanocrystals. Nanoscale size reduction, order–disorder phase transition and band gap evolution tailor the thermoelectric properties in AgCuS.

## 1. Introduction

Investigations of the structural phase transition in inorganic materials are not only of fundamental interest in solid state inorganic chemistry but are also useful for advanced technological applications.^1^,^2^ In addition to such phase transitions involving changes in the atomic configuration, many of the solids undergo an orientation change of the electron clouds, influencing the state of an electron spin on passing through the phase transition. The change in crystal structures, electron clouds and electron spin states can result in changes in the electronic structure and phonon dispersion of the materials. During phase transitions, an increase or decrease of the band gap of a material can give rise to insulator or metallic states, respectively, which then controls the carrier concentration and the electronic properties.^1^,^3^ Phase transitions in solids lead to many unusual and important properties such as superconductivity,^4^ superionic conduction,^5^–^8^ optical storage,^9^ the photoelectronic effect,^10^ the giant magnetocaloric effect,^11^ giant magnetoresistance,^12^ p–n–p or p–n type conduction switching,^12^–^14^ and thermoelectricity.^15^,^16^ Design and control of the changes in the physical properties associated with a phase transition have been key to the development of current functional materials.^17^,^18^

Noble metal based binary, ternary or quaternary silver and copper chalcogenides, chalco-halides, and halides are a special class of semiconductor, which show intriguing phase behavior, structural variability, a high degree of disorder and high ion dynamics.^6^,^13^,^14^ These compounds are made of weakly coupled cationic and anionic substructures.^6^,^7^,^18^ Most of the compounds of this class undergo superionic structural phase transitions with temperature, which involve changes in the substructure of their mobile ions. This class of compounds generally shows macroscopic movement of the Ag^+^/Cu^+^ ions in their superionic phase as cationic lattice sites are accessible to them with only a small energy barrier, while the chalcogen or chalco-halogen sublattice remains rigid.^6^,^18^ Recently, applications of these compounds have been explored in thermoelectrics, solar energy conversion and resistive switching.^14^–^20^ Ag_10_Te_4_Br_3_, AgBiSe_2_ and AgCuS exhibit reversible p–n–p type conduction switching as a function of temperature, which can have potential applications in temperature controlled/sensitive diodes and transistors.^13^,^14^,^18^,^19^,^21^ The p–n–p type conduction switching is highly sensitive to the order–disorder phase transition, evolution of the electronic structures during the phase transition and the carrier type of the material.^17^,^18^ Substitution of foreign cations or anions can modulate the electronic structure and carrier transport in a material, which may tune the phase transition temperature. Thus, anion substituted Ag_10_Te_4_Br_2.8_I_0.2_ shows a shift of the p–n–p conduction switching temperature towards room temperature, but the change in the Seebeck coefficient was smaller than that of pristine Ag_10_Te_4_Br_3_.^21^ It must be mentioned that AgBiSe_2_ shows p–n–p type conduction switching in its nanocrystalline form,^19^ but its bulk counterpart does not exhibit such conduction type switching.16*a* Thus the particle or grain size has an important role in the conduction switching property as the electronic band gap of a material is highly sensitive to the crystallite size. Hence, it would be interesting to study the effect of the dimensionality of the material on the conduction switching property and the phase transition temperature^22^ of inorganic materials.

Recently, we have discovered that bulk polycrystalline AgCuS exhibits a reversible p–n–p type conduction switching along with a colossal change in its Seebeck value (Δ*S* of ∼1757 μV K^–1^) at *T* ∼364 K during the first superionic (β–α) phase transition.^13^ It should be noted that previous research has been solely focused on the bulk AgCuS synthesized by solid state reaction at high temperature,^13^,^18^ but there is no report on the investigation of the thermoelectric properties and order–disorder phase transition of AgCuS in its nanocrystalline form.

Herein, we present a facile and room temperature solution based synthesis for capping free AgCuS nanocrystals and investigate their temperature dependent (300–550 K) structural phase transition and thermoelectric properties. The synthesis process is very simple and effective with a high yield (∼90%). Temperature dependent synchrotron powder X-ray diffraction, Raman spectroscopy and heat capacity measurement indicates the observation of two superionic phase transitions, from a room temperature ordered orthorhombic (β) to a partially disordered hexagonal (α) at ∼365 K and from the hexagonal phase (α) to a fully disordered cubic phase (δ) at ∼439 K, in nanocrystalline AgCuS. A sharp decrease of the Seebeck coefficient value from 783 μV K^–1^ at 360 K to 418 μV K^–1^ at 367 K is observed during the orthorhombic to hexagonal (β–α) phase transition in AgCuS nanocrystals obtained after 30 min reaction. We found that, unlike its bulk counterpart, nanocrystalline AgCuS does not exhibit p–n–p type conduction switching during the first superionic phase transition. Nanocrystalline AgCuS exhibits a higher electronic band gap (1.2 eV) compared to that of its bulk counterpart (0.9 eV) and the Raman spectroscopy shows the absence of Cu–S bond vibration during the (β–α) transition, which collectively disfavor the creation of a semimetallic intermediate electronic state during the orthorhombic (β) to hexagonal (α) phase transition that was responsible for the p–n–p type conduction switching in bulk AgCuS.^13^ Furthermore, temperature dependent positron annihilation spectroscopy indicates that a Ag vacancy is responsible for the p-type conduction and the localized nature of Ag vacancies in AgCuS nanocrystals disfavors the macroscopic movement of Ag/Cu during the order–disorder phase transition that was responsible for the p–n–p type conduction switching in bulk AgCuS.^13^

## 2. Experimental section

### Materials

Copper(ii) nitrate trihydrate (Alfa Aesar, 99%), silver(i) nitrate (Sigma Aldrich, 99%), sulfur powder (Alfa Aesar, 99.999%) and sodium borohydride (Aldrich, 98%), and tetraethylene glycol (Sigma Aldrich, 99%) were used for synthesis without further purification.

### Synthesis of AgCuS nanocrystals

Capping agent free AgCuS nanocrystals were synthesized by a bottom up wet chemical method. At first, silver nitrate (100 mg, 0.59 mmol), copper nitrate (142.2 mg, 0.59 mmol), sulfur powder (18.9 mg, 0.59 mmol) and tetraethylene glycol (15 ml) were placed in a round bottom flask, then the reaction mixture was sonicated for 15 minutes to get a well dispersed solution. NaBH_4_ (66.8 mg, 1.76 mmol) was added to the reaction mixture with stirring at room temperature (300 K) under N_2_ atm. By variation of the reaction time, we were able to synthesize nanocrystals of different sizes. Nanocrystals of ∼40 nm and ∼120 nm were synthesized with a reaction time of 30 min and 12 hours respectively. After the reaction the black colored product was isolated by centrifugation. The AgCuS nanocrystal powder was washed several times with ethanol to remove surface adsorbed tetraethylene glycol and was finally dried under a vacuum at 60 °C for 2 h. The yield of the reaction is ∼90%.

### Thermogravimetric analysis (TGA)

TGA was performed using a TGA/DSC 2 STAR instrument in the temperature range of 300–730 K under nitrogen atmosphere with a ramp rate of 5 K min^–1^.

### Fourier transform infrared spectroscopy (FTIR)

A Bruker IFS 66v/S spectrometer was used for recording the FTIR spectra.

### Powder X-ray diffraction

Room temperature powder X-ray diffraction of the samples was recorded using Cu K_α_ (*λ* = 1.5406 Å) radiation on a laboratory Bruker D8 diffractometer. Temperature dependent synchrotron powder X-ray diffraction measurements were carried out under N_2_ flow with an X-ray beam of *λ* = 1.0279 Å, at BL-18B (Indian beamline), Photon Factory, KEK, Tsukuba, Japan. The energy of the beam was set by a Si(111) double crystal monochromator, which was cross checked with a Si (640b NIST) standard. All the measurements were carried out in Bragg–Brentano geometry with a divergence slit (300 μm), an anti-scattering slit (350 μm) and a receiving slit (300 μm). High temperature measurements were carried out with Anton Paar DHS 1100 heat cell.

### Field emission scanning electron microscopy

FESEM imaging has been performed using a NOVA NANO SEM 600 (FEI, Germany) operated at 15 kV. Energy dispersive spectroscopy (EDAX) analysis has been performed with an EDAX Genesis instrument attached to the FESEM column.

### Transmission electron microscopy

The TEM experiment has been done using a JEOL (JEM3010) TEM fitted with a Gatan CCD camera operating at 300 kV accelerating voltage and also using a FEI TECNAI G^2^ 20 STWIN TEM operating at 200 kV. EDAX elemental mapping was performed during STEM imaging. Background was subtracted (using multi-polynomial model) during the data processing for EDAX elemental mapping (with 500 eV minimum region of interest width).

### Inductively coupled plasma atomic emission spectroscopy

ICP-AES measurements were carried out using a Perkin-Elmer Optima 7000DV instrument. ICP-AES measurements were carried out by dissolving nanocrystals in aqua regia (HNO_3_ : HCl = 1 : 3) followed by diluting with milipore water. Ag standard (1000 mg L^–1^, Merck), Cu standard (1000 mg L^–1^, Fluka) and S standard (1000 mg L^–1^, Sigma-Aldrich) were used to determine the compositions in ICP.

### X-ray photoelectron spectroscopy

XPS measurements were performed with a Mg-Kα (1253.6 eV) X-ray source with a relative composition detection better than 0.1% on an Omicron Nanotechnology spectrometer.

### Differential scanning calorimetry

A Netzsch DSC 200F3 was used for the DSC measurements with a heating/cooling rate of 20 K min^–1^ between 290 and 550 K in N_2_ atmosphere.

### Raman spectroscopy

Temperature-dependent (300–473 K) Raman spectroscopy measurements were carried out with a LABRAMHR spectrometer in N_2_ atmosphere. The excitation wavelength of the laser was 514 nm.

### Seebeck coefficient and electrical conductivity

In order to measure their thermoelectric properties, the AgCuS nanocrystals were pressed at room temperature into a rectangular bar (2 × 2 × 8 mm^3^) using an AIMIL die-press at 60 kN m^–2^ pressure. The rectangular shaped sample was sealed in a quartz tube under vacuum (10^–5^ Torr) and then sintered for 3 h at 573 K which is above the upper limit of the thermoelectric measurement temperature. We could achieve a density of ∼94%, which is really good as the present nanoparticles do not contain any capping agents. The FESEM image after sintering indicates that the sample became more compact and the adjacent nanoscale grains are closely attached to each other with a slight increase in the grain size (Fig. S3, ESI†). Electrical conductivity and the Seebeck coefficient were measured under a helium atmosphere from room temperature to about 550 K on a ULVAC-RIKO ZEM-3 instrument system.

### Hall measurement

Room temperature carrier concentration has been derived from the Hall coefficient measurement using a PPMS (Physical Property Measurement System, Quantum Design, USA).

### Band gap measurement

To probe the optical energy gap, optical diffuse reflectance measurements were performed on the nanocrystalline powder sample at room temperature. The spectra were recorded at the range of 600 nm to 2400 nm using a Perkin Elmer Lambda 900, UV/Vis/NIR spectrometer. Absorption (*α*/*Λ*) data was calculated from the reflectance data using the Kubelka–Munk equation: *α*/*Λ* = (1 – *R*)^2^/(2*R*), where *R* is the reflectance and *α* and *Λ* are the absorption and scattering coefficients, respectively. The energy band gap was derived from an *α*/*Λ* (a.u.) *vs. E* (eV) plot.

### Positron annihilation spectroscopy

Positron annihilation lifetime has been measured with a fast–fast coincidence assembly consisting of two constant fraction differential discriminators (Fast ComTech Model number 7029A). The detectors are 25 mm-long × 25 mm tapered to 13 mm diameter cylindrical BaF_2_ scintillators optically coupled to Philips XP2020Q photomultiplier tubes. The resolving time (full width at half maximum, FWHM), of the present fast–fast coincidence assembly is ∼220 ps. For the present positron annihilation measurement, a 10 μCi ^22^Na source of positrons (enclosed between 1.5 μm thin nickel foils) has been sandwiched between two identical and plane faced pellet type AgCuS nanocrystalline samples. The measured positron annihilation lifetime spectrum has been analyzed by computer programme PATFIT-88.^23^ The source component^24^ has been evaluated by measuring a positron lifetime spectrum of 99.9999% pure Al and properly subtracted from the sample spectrum. For the Doppler broadening of the positron annihilation radiation (DBPAR) measurement, a HPGe detector (efficiency: 12%; type: PGC 1216sp of DSG, Germany) with an energy resolution of 1.1 keV at 514 keV of ^85^Sr has been used for the detection of the 511 keV γ-ray. The DBAR spectrum has been recorded in a dual ADC based – multiparameter data acquisition system (MPA-3 of FAST ComTec, Germany). For the temperature dependent DBPAR measurement, the source sample sandwich has been placed inside a furnace (30–600 °C with ±2 °C at the sample site).^25^ For each temperature, about 10^7^ counts have been recorded in a dual ADC based multiparameter data acquisition system (MPA-3 of FAST ComTec., Germany). The Doppler broadening of the annihilation 511 keV γ-ray spectrum has been analyzed by evaluating the conventional line-shape parameters (S-parameter). The S-parameter is calculated as the ratio of the counts in the central area of the 511 keV photo peak (|511 keV – *E*_γ_| ≤ 0.85 keV) and the total area of the photo peak (|511 keV – *E*_γ_| ≤ 4.25 keV).^25^ The S-parameter represents the fraction of positrons annihilating with the lower momentum electrons with respect to the total electrons annihilated.

## 3. Results and discussion

**Fig. 1:**
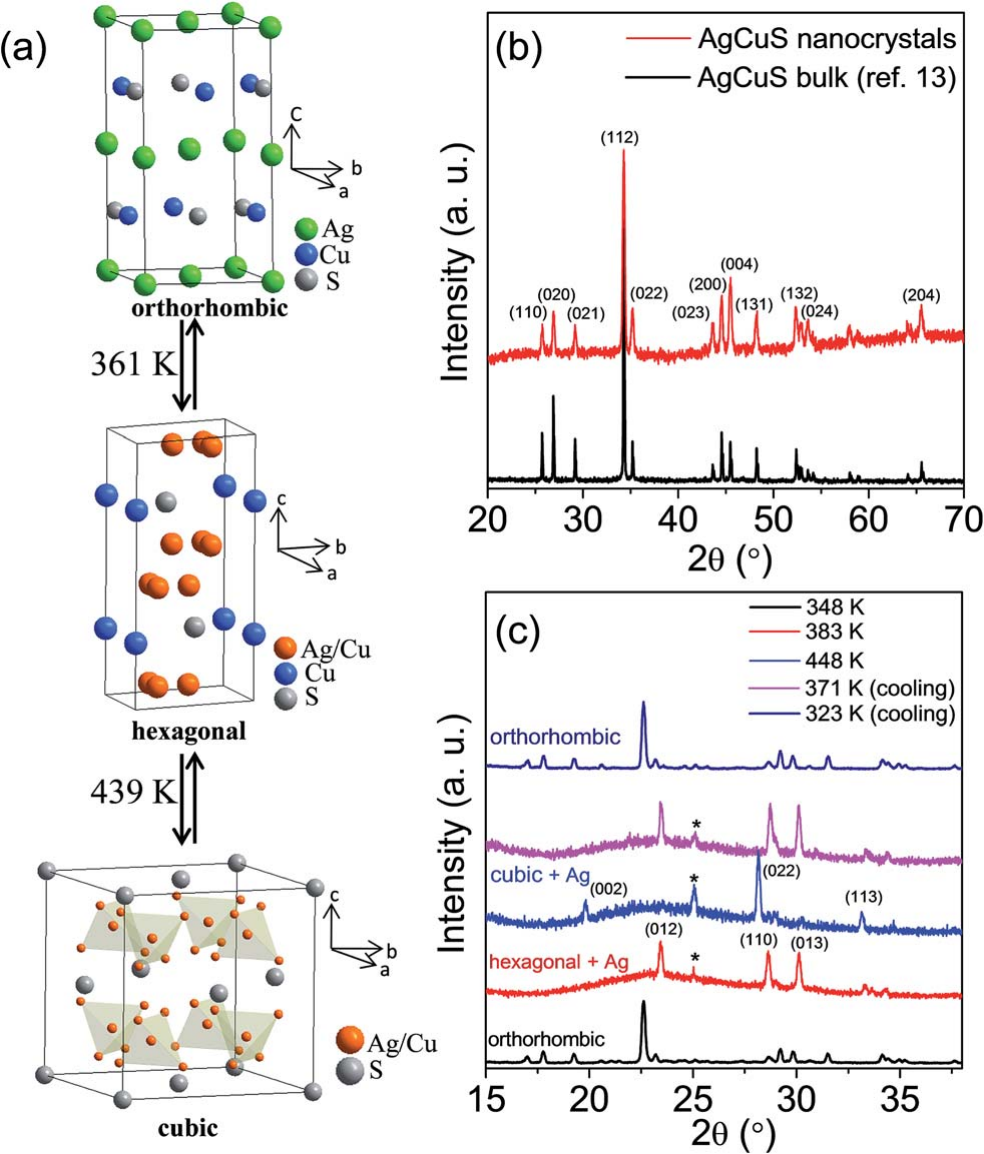
(a) Crystal structure evolution of AgCuS with temperature. In the hexagonal phase, Ag and Cu are partially disordered in 12k position. In the cubic structure, Ag and Cu are disordered in 8c and 32f positions.^26^ (b) Powder XRD pattern of AgCuS nanocrystals (obtained after 30 min of reaction) measured at lab source (Cu K_α_; *λ* = 1.5406 Å). The powder XRD pattern of bulk AgCuS was given for comparison.^13^ (c) Temperature dependent (300–448 K) heating/cooling cycle synchrotron (*λ* = 1.0279 Å) powder X-ray diffraction patterns of AgCuS nanocrystals obtained after 30 min of reaction.

AgCuS is a polymorphous semiconductor that shows a superionic phase transition with changing temperature (Fig. 1a).^13^,^26^,^27^ At room temperature, AgCuS crystallizes in an ordered orthorhombic β-AgCuS phase (space group, *Cmc*2_1_). The β-AgCuS crystal structure consists of distorted hexagonal close packing (hcp) of sulfur atoms, and in the hcp sulfur layer three coordinated Cu atoms are occupied (Fig. 1a). The planes parallel to (001) are made up of layers of loosely packed face-centered Ag atoms alternating with that of triangularly coordinated sulfur and copper atoms (Fig. 1a).^26^,^27^ At high temperature, β-AgCuS undergoes two structural phase transitions, first to a partially cation disordered superionic hexagonal α-AgCuS phase (space group *P*6_3_/*mmc*) at 361 K, and finally at 439 K to a fully cation disordered superionic cubic δ-AgCuS phase (space group *Fm*3*m*).^13^,^26^,^27^ In the first superionic α phase, sulfur atoms conserve the hcp sublattice, but the Ag atoms are in a fully disordered state and 75% of the Cu atoms reside in the ordered position (Fig. 1a). In the second high temperature superionic δ phase, sulfur atoms still remain ordered and form a rigid fcc lattice, in which Ag/Cu are randomly distributed at tetrahedral and octahedral interstitial sites (Fig. 1a).^13^,^26^,^27^

**Fig. 2:**
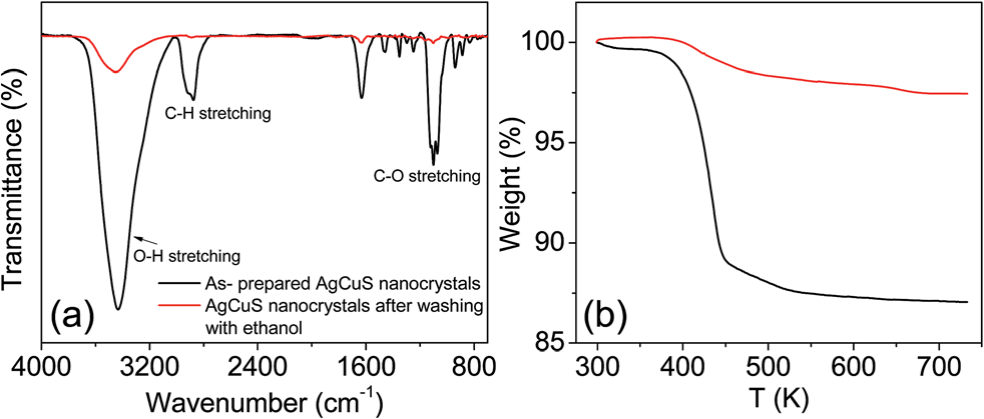
(a) FTIR spectra and (b) TGA curve of AgCuS nanocrystals (∼40 nm), before and after washing with ethanol.

Capping agent free nanocrystals of AgCuS were synthesized using a simple and room temperature wet chemical synthesis method. Nitrate salts of Ag^+^ and Cu^+^ were used as the metal precursors, and sulfur powder was used as a sulfur source. NaBH_4_ was used for the reduction of the metal nitrate salts. Tetraethylene glycol (TEG) was used as the solvent. TEG is a short chain aliphatic organic compound which contains two hydroxyl groups (–OH group) on the terminal carbon. Due to the presence of two terminal –OH groups, it can adsorb on the reactive surface of the as synthesized nanocrystal. In order to remove the surface adsorbed TEG, we have repeatedly washed the sample with ethanol (TEG is soluble in ethanol) and finally dried it under vacuum at 60 °C for 2 h. FTIR and TGA measurements have been used to probe the removal of the TEG after washing with ethanol (Fig. 2). The presence of strong peaks in the FTIR spectra due to the vibration of –O–H, C–H and C–O bonds in the synthesized AgCuS nanocrystals indicates the presence of TEG in the sample (Fig. 2a). However, significant reduction of the peak intensities was observed after washing with ethanol, indicating most of the TEG was removed from the sample (Fig. 2a). TGA measurements also confirm the removal of surface adsorbed TEG from the as synthesized sample as the weight loss decreases from ∼12% to ∼1.5% in the as synthesized and ethanol treated samples, respectively (Fig. 2b). Similar types of result were earlier observed in ambient synthesized Cu_2_Se nanostructures.20*c* The AgCuS nanocrystals were characterized using powder X-ray diffraction (XRD), X-ray photoelectron spectroscopy (XPS), energy dispersive X-ray analysis (EDAX), inductively coupled plasma atomic emission spectroscopy (ICP-AES), scanning and transmission electron microscopy (FESEM/TEM), and selected area electron diffraction analysis (SAED).

Room temperature PXRD (Cu K_α_, *λ* = 1.5406 Å) of the as synthesized nanocrystals could be indexed based on the orthorhombic β-AgCuS phase (space group, *Cmc*2_1_) (Fig. 1b and S1, ESI†). In order to understand the structural evolution with temperature, we have performed high temperature (300–448 K) heating/cooling cycle synchrotron powder XRD of the AgCuS nanocrystals (obtained after 30 min of reaction) (Fig. 1c). Temperature dependent PXRD data shows a clear structural phase transition from room temperature orthorhombic (β) to the hexagonal (α) phase around 365 K. With further heating the hexagonal (α) phase transforms to the cubic phase (δ). The phase transformation is reversible in nature as confirmed by the cooling cycle PXRD data. Interestingly, we have observed the appearance of a little metallic Ag phase (* marked in Fig. 1c) along with the α- and δ-AgCuS phase on heating the β-AgCuS nanocrystalline sample, which then disappeared on cooling back to the orthorhombic β-AgCuS. This was not observed in the case of the bulk AgCuS sample,^13^ which we will discuss in a later section.

**Fig. 3:**
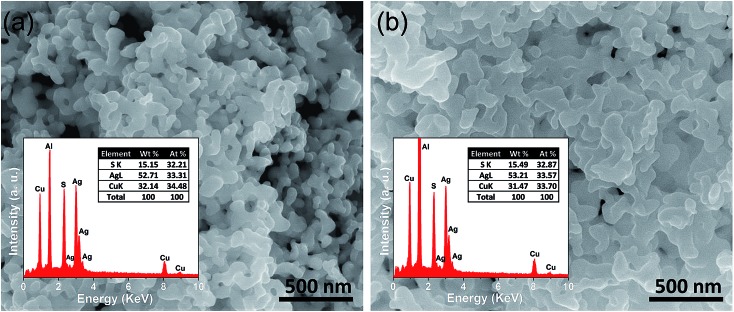
FESEM images of nanocrystalline AgCuS obtained after the reaction times of (a) 30 min and (b) 12 h, respectively. Insets in (a) and (b) show EDAX spectra of respective nanocrystal with elemental percentage.

FESEM images of the as synthesized sample indicate that AgCuS nanoparticles are agglomerated in nature. Absence of an additional capping agent during the synthesis results in the agglomeration of the AgCuS nanoparticles. The FESEM image indicates the average particle size of the nanocrystals obtained after 30 min of the reaction is ∼40 nm (Fig. 3a). The average particle size of the nanocrystals obtained after 12 h of reaction is ∼120 nm (Fig. 3b) and particles becomes more agglomerated and interconnected with increasing reaction time. EDAX analysis indicates the presence of Ag, Cu and S in the proper ratios (insets of Fig. 3). ICP-AES measurements also indicate the composition of nanocrystals to be Ag_0.97_CuS_0.98_, which is close to the nominal composition.

**Fig. 4:**
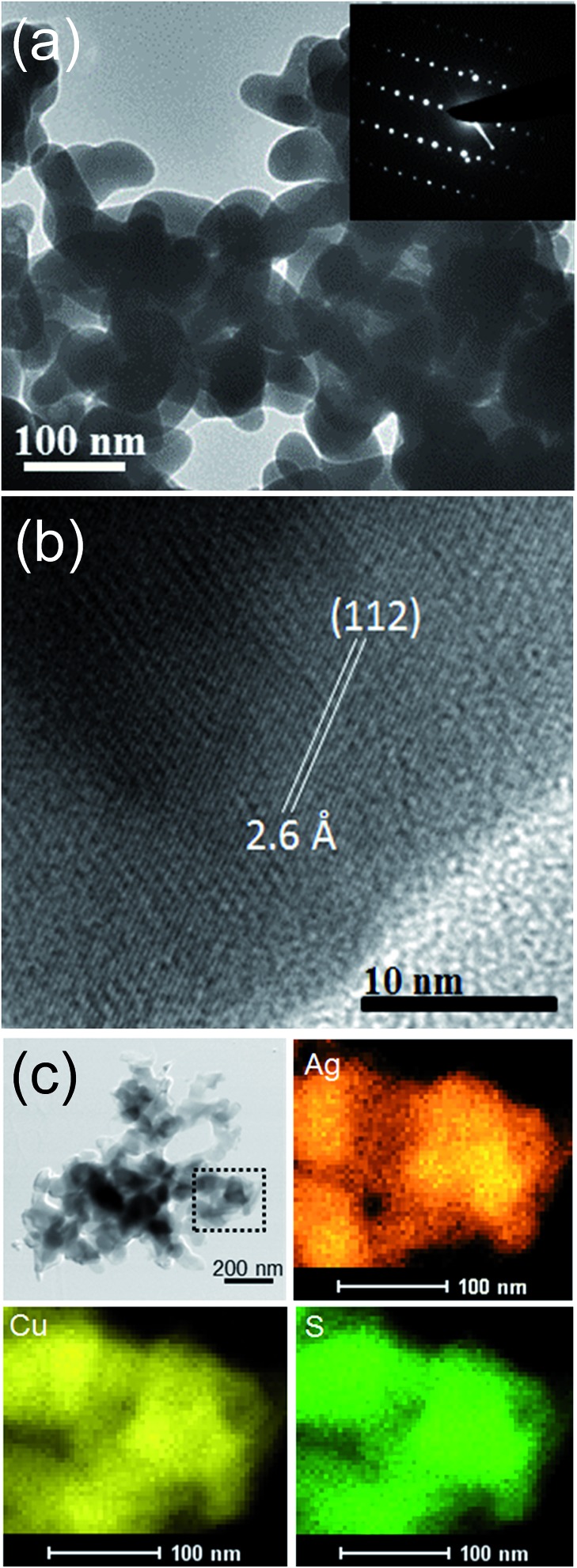
(a) TEM image and inset in (a) shows the SAED pattern, (b) HRTEM image of AgCuS nanocrystals (30 min reaction). (c) STEM image of and STEM-EDAX compositional mapping over a group of nanocrystals (from the highlighted portion of the STEM image).

The TEM image of the AgCuS nanocrystals obtained after 30 min of reaction also indicates the agglomerated nature of the particles (Fig. 4a). The SAED pattern taken from a particle indicates a single crystalline nature of the AgCuS nanocrystal (inset of Fig. 4a). The high resolution TEM (HRTEM) image shows a clear lattice spacing of 2.6 Å corresponding to the (112) inter planar distance of orthorhombic AgCuS (Fig. 4b). Additionally, we have performed EDAX elemental mapping analysis during the scanning transmission electron microscopy (STEM) investigation. In Fig. 4c we present a STEM image of the nanocrystals obtained after 30 min of reaction. The STEM-EDAX compositional mapping over the group of nanocrystals indicates the single phase homogeneity of the AgCuS nanocrystals.

In order to further confirm the presence of the relevant elements and their oxidation states, we have performed XPS of the nanocrystalline AgCuS (Fig. S2, ESI†). The presence of two strong peaks at 373.5 eV and 367.4 eV in the XPS is due to Ag(i) 3d_3/2_ and Ag(i) 3d_5/2_, respectively (Fig. S2b, ESI†). The peaks at 952 eV and 932.2 eV correspond to Cu(i) 2p_1/2_ and Cu(i) 2p_3/2_ respectively. An additional small peak centered at 942.6 eV was evidenced, which is due to the presence of Cu(ii) (Fig. S2c, ESI†). As the synthesized nanocrystals are capping free, surface oxidation of the AgCuS nanocrystals resulted in the formation of a minor amount of the more stable Cu(ii), which was otherwise not detected by PXRD. A peak located at 161 eV is due to S 2p (Fig. S2d, ESI†).

**Fig. 5:**
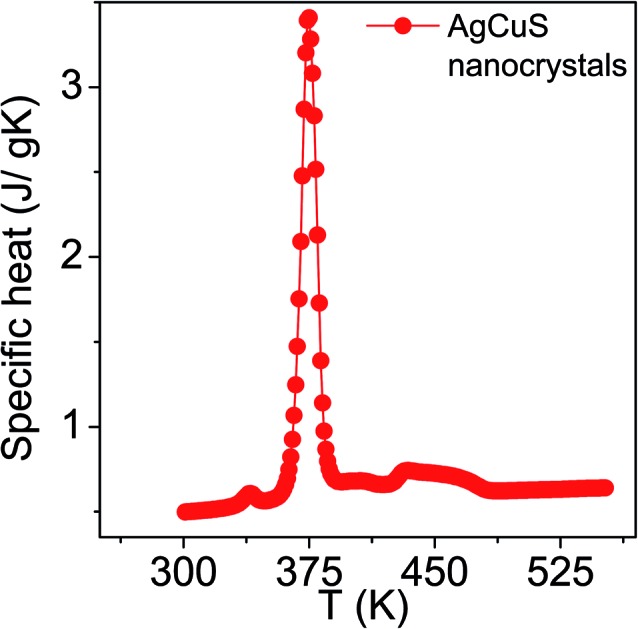
Temperature dependent specific heat (*C*_p_) of nanocrystalline AgCuS obtained after 30 min of reaction.

The temperature dependent specific heat (*C*_p_) of nanocrystalline AgCuS obtained after 30 min of reaction was measured using a differential scanning calorimetry (DSC) technique and demonstrates two phase transitions above room temperature (Fig. 5). A λ-shaped peak centered at ∼375 K corresponds to the phase transition from the ordered orthorhombic (β) to the partially disordered hexagonal (α) phase. A broad peak centered at ∼445 K corresponds to the phase transition from the partially disordered hexagonal (α) to the fully disordered cubic (δ) phase. A similar observation has also been evidenced in the bulk AgCuS sample.^13^ However, the additional peak at ∼408 K corresponding to the phase boundary of the biphasic hexagonal–cubic region was present for the bulk AgCuS sample,^13^ which is absent in the case of the nanocrystalline AgCuS sample. This indicates that the hexagonal (α) phase transforms to the cubic (δ) phase without passing through a mixture of hexagonal (α) + cubic (δ) phases in the present nanocrystalline AgCuS sample, which is consistent with the temperature dependent PXRD data (Fig. 1c). The (β–α) transition takes place from an ordered state to a partially disordered state (onset of order–disorder transition) with a significant change in volume of 2.3%, while the (α–δ) transition occurs from a partially disordered state to a fully disordered state with a volume change of 0.6%.^13^,^26^ The above discussion explains why the anomalies in the different measurements (Seebeck and *C*_p_) are generally dramatic and sharp during the (β–α) transition compared to that of an (α–δ) transition.

**Fig. 6:**
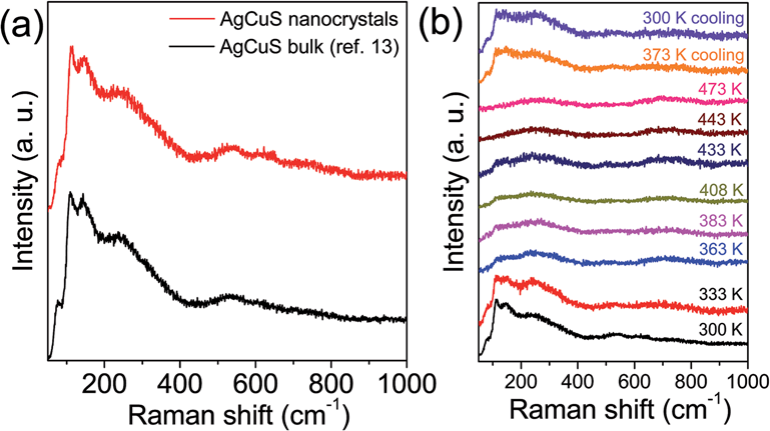
(a) Room temperature Raman spectra of nanocrystalline AgCuS (obtained after 30 min of reaction) compared with that of the previously reported bulk AgCuS sample.^13^ (b) Temperature dependent Raman spectra of nanocrystalline AgCuS (obtained after 30 min of reaction).

In Fig. 6a, we present the room temperature Raman spectrum of nanocrystalline AgCuS (obtained after 30 min of reaction) which is compared with its bulk counterpart. All of the peaks in the Raman spectrum of the nanocrystalline sample are matching with that of bulk AgCuS. A broad peak at ∼240 cm^–1^ can be assigned to the Ag–S bond vibration.^13^,^28^,^29^ The peaks below ∼150 cm^–1^ can be assigned to Ag/Cu lattice vibration.^13^,^30^ Temperature dependent Raman spectra of nanocrystalline AgCuS were presented in Fig. 6b. During the (β–α) transition temperature (363 K), the peaks associated with the Ag lattice vibration completely disappear as the Ag atoms begin to disorder in the hexagonal (α) phase. However, the most interesting observation is that the peak associated with the Cu–S bond vibration at ∼474 cm^–1^ is absent in the nanocrystalline AgCuS during the (β–α) transition, which was present in the bulk AgCuS sample (Fig. S4, ESI†). Previously, using density functional theory (DFT) based calculations, we have shown that the Cu–S hybridized orbital plays a crucial role in the p–n–p type conduction switching of bulk AgCuS, as it contributes to the semimetallic intermediate state during the (β–α) transition.^13^ Absence of the Cu–S bond vibration results in a significant change in the conduction property of the nanocrystalline AgCuS, which will be discussed in a later part of the paper. Further increasing the temperature to 443 K, the cubic (δ) phase does not show any Raman signal due to the absence of macroscopic polarizability as the Ag/Cu are fully disordered.^13^ The phase transitions are reversible in nature, which is confirmed by taking the Raman spectra on cooling.

**Fig. 7:**
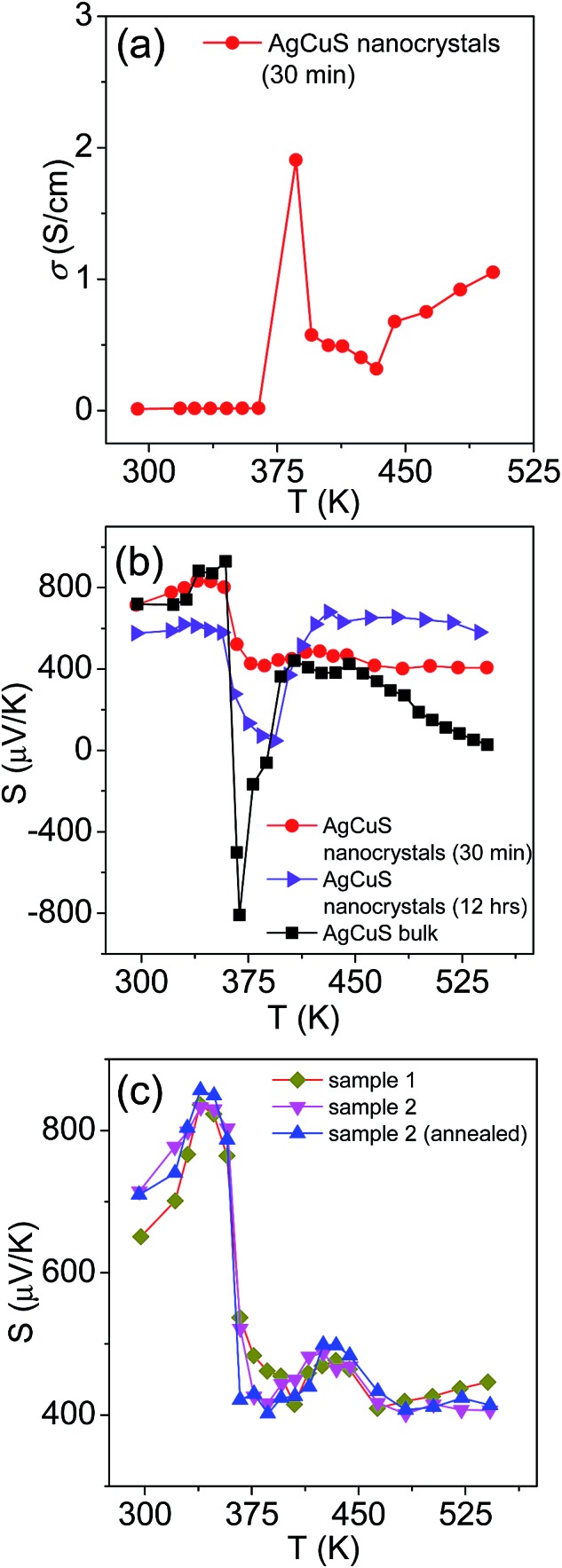
(a) Temperature dependent electrical conductivity (*σ*) of AgCuS nanocrystals obtained after 30 min of reaction. (b) Temperature dependent Seebeck coefficients (*S*) of AgCuS nanocrystals obtained after 30 min and 12 h of reaction, which are compared to that of their bulk counterpart.^13^ (c) Temperature dependent Seebeck coefficients (*S*) of different batches of nanocrystalline AgCuS samples (30 min of reaction) show the reproducibility and temperature stability.

In Fig. 7, we present the temperature dependent thermoelectric properties for nanocrystalline AgCuS. Temperature dependent electrical conductivity (*σ*) indicates a sharp anomaly during the (β–α) transition (∼361 K); while the increase in *σ* during the (α–δ) transition (∼433 K) is not very significant (Fig. 7a). The trend of temperature dependent *σ* in the case of nanocrystalline AgCuS (obtained after 30 min of reaction) is quite similar to that for the bulk AgCuS.^13^ However, the value of *σ* for the nanocrystalline sample is higher compared to that of bulk AgCuS (Fig. S5, ESI†), which is due to the higher carrier concentration in the nanocrystalline phase (*n*_nano_ = 2.6 × 10^15^ cm^–3^ at 300 K) than in its bulk counterpart (*n*_bulk_ = 1.3 × 10^15^ cm^–3^ at 300 K). Higher p-type carrier concentration in the nanocrystalline AgCuS sample is due to the formation of more Ag vacancies in the nanoscale, which act as a p-type dopant and inject more positively charged holes into the nanocrystalline sample.

Nanocrystalline AgCuS (obtained after 30 min of reaction) shows a positive sign Seebeck coefficient (*S*) in the temperature 300–550 K range, indicating p-type conduction (Fig. 7b). An *S* value of ∼710 μV K^–1^ is measured at room temperature, which increases to 855 μV K^–1^ at 350 K. A sharp decrease in *S* from ∼783 μV K^–1^ at 360 K to ∼418 μV K^–1^ at 367 K is observed during the orthorhombic to hexagonal (β–α) phase transition (Fig. 7b). The decrease in *S* during the (β–α) phase transition temperature is consistent with the sharp increases in *σ* at ∼361 K. We have compared the temperature dependent *S* data of the AgCuS nanocrystals to the previously reported bulk AgCuS in Fig. 7b.^13^ Bulk AgCuS is known to show reversible p–n–p type conduction switching with a large change in the Seebeck coefficient (Δ*S* = ∼1757 μV K^–1^) during the orthorhombic to hexagonal (β–α) phase transition.^13^,^18^ Interestingly, we have not observed any p–n–p type conduction switching after the size reduction to the nanoscale in AgCuS. We observe a slight dip in temperature dependent *S* during the hexagonal to cubic (α–δ) phase transition at 437 K (Fig. 7b). Measurements on the different batches of samples and an annealed sample reproduced a similar trend in the temperature dependent *S* data for AgCuS nanocrystals (Fig. 7c). Heating–cooling cycle *S* measurements indicate the reversibility of the phase transition. The repeated heating–cooling cycle measurements on the same sample after annealing (573 K for 12 h) also show the reversibility of the transition and hence confirm the temperature stability of the thermoelectric properties above the phase transition temperature (Fig. S6, ESI†). It must be mentioned that the heating–cooling cycle Seebeck data shows small hysteresis during the (β–α) and (α–δ) phase transitions (Fig. S6, ESI†). Superionic phase transitions in AgCuS involve a change in volume of 2.3% and 0.6% during the β–α and α–δ phase transitions, respectively.^13^,^26^ The phase transitions in AgCuS are first order type as the volume (*V*) is a first derivative of Gibbs free energy (*G*), 
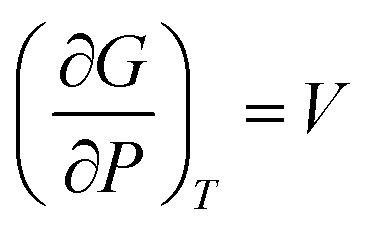
, where, *P* is pressure and *T* is temperature. First order phase transitions are generally associated with a hysteresis due to the change in volume during the transition.^1^ A change in volume during the phase transition might result in the small hysteresis in the temperature dependent Seebeck data. Thus, the bump on the heating cycle transformed to a small dip on cooling during the (α–δ) phase transition (Fig. S6, ESI†).

We have also performed the temperature dependent *S* measurement on the nanocrystals obtained after 12 h of reaction. Larger sized (∼120 nm) nanocrystalline AgCuS also shows p-type conduction in the 300–550 K range, but interestingly during the phase transition (β–α) the *S* value decreases to a much lower value (∼133 μV K^–1^ at 374 K) than that for the smaller nanocrystals (∼40 nm) obtained after 30 min of reaction (Fig. 7b). *S vs. T* data of the different particle sized AgCuS nanocrystals indicate that with increasing reaction time (*i.e.* increasing particle size) the conduction properties of the system are moving towards the conduction properties of bulk AgCuS.

The temperature dependent thermoelectric figure (*ZT*) of merit was estimated from the thermoelectric data, which has been presented in Fig. S7 (ESI†). The nanocrystalline AgCuS sample shows a higher *ZT* value than that of the bulk sample due to higher electrical conductivity and lower thermal conductivity values.

**Fig. 8:**
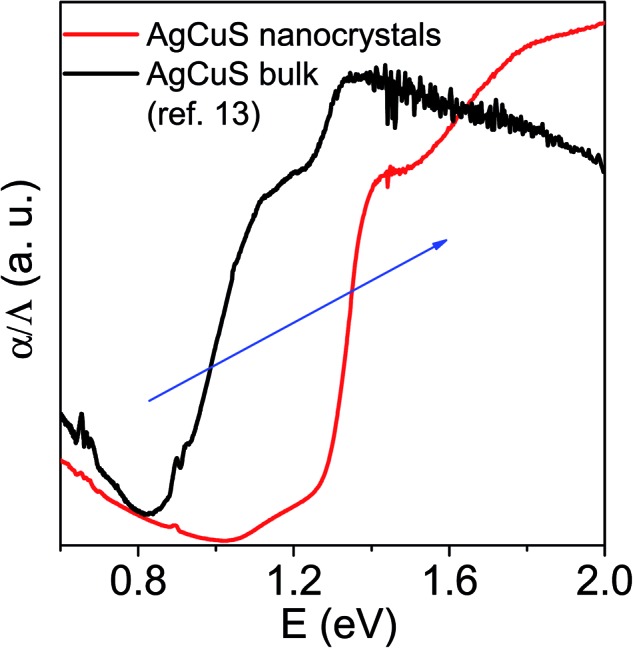
Room temperature optical absorption spectra of as synthesized AgCuS nanocrystals (obtained after 30 min of reaction) compared with the previously reported bulk AgCuS.^13^

In order to further understand the thermoelectric properties of the AgCuS nanocrystals, we have performed optical band gap and temperature dependent positron annihilation spectroscopy measurements. Structural phase transformation in solids can result in a decrease of the electronic band gap and hence sometimes creates a semimetallic intermediate state where the Fermi level can reshuffle from the valence to the conduction band, thus giving rise to a conduction switching property.^13^,^14^,^18^,^19^ Previously, first principle density functional theoretical calculations on the electronic structure of bulk AgCuS showed that closing of the band gap occurred during the orthorhombic (β) to hexagonal phase (α) transition. This caused the formation of an intermediate semimetallic state, which was responsible for the p–n–p conduction switching in bulk AgCuS. The spectroscopically measured band gap of the nanocrystalline AgCuS (obtained after 30 min of reaction) is ∼1.2 eV at room temperature (Fig. 8), which is higher comparatively than that of the bulk AgCuS (∼0.9 eV). We believe that the increase in the band gap is one of the primary reasons for the absence of conduction type switching in nanocrystalline AgCuS. The higher electronic band gap in nanocrystalline AgCuS compared to that of its bulk counterpart is not sufficient for closing of the gap between the conduction and valence band (*i.e.* formation of semimetallic electronic state) during the orthorhombic (β) to hexagonal (α) phase transition. Fig. S8 (ESI†) represents a schematic of changes of the band gap of bulk and nanocrystalline AgCuS during the first phase transition.

**Fig. 9:**
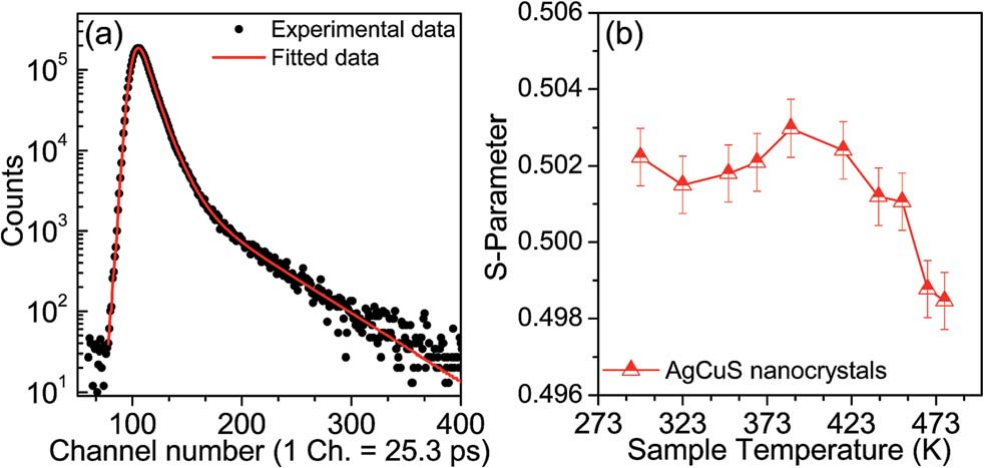
(a) Room temperature positron annihilation lifetime spectrum for the AgCuS nanocrystals (obtained after 30 min of reaction). Solid red line shows the fitting for the determination of positron life time components (b) Temperature dependent Doppler broadening S-parameter of nanocrystalline AgCuS (30 min of reaction).

Positron annihilation spectroscopy is a powerful technique for the detection of vacancies or defect types in solids. In Fig. 9a, we present a room temperature positron annihilation lifetime spectrum of the nanocrystalline AgCuS (obtained after 30 min of reaction). The annihilation lifetime spectrum for the nanocrystalline AgCuS sample (variance of fit < 1 per channel) yields three lifetime components (Table 1), which are similar to those previously reported for bulk AgCuS.^13^ The shortest lifetime component (*τ*_1_) has been assigned to the free annihilation of positrons,^31^ while the intermediate lifetime component (*τ*_2_) is due to the annihilation of positrons in the defect site, mainly the Ag vacancy.^13^,^19^ The long lifetime component (*τ*_3_) originates from the formation of positronium at the sample surface or in the large voids inside the sample. Since the nanocrystal size (∼40 nm) is less than the positron diffusion length, the positrons are mainly trapped in the vacancy like defects at the surfaces/grain boundaries. Here the grain surface is enriched with Ag vacancy type defects, thus *τ*_1_ is an admixture with the positron lifetime in Ag mono-vacancy type defects present at the grain boundaries. The intermediate lifetime component is due to the trapping of positrons in Ag vacancy clusters. Positron annihilation lifetime measurements on the nanocrystalline AgCuS clearly show that the nanocrystalline surface is enriched with Ag vacancy like defects and defect clusters. This finding indicates that Ag vacancies are indeed responsible for the p-type conduction in nanocrystalline AgCuS.

**Table 1:** Positron lifetime components of AgCuS nanocrystals

*τ*_1_ (ps)	*I*_1_ (%)	*τ*_2_ (ps)	*I*_2_ (%)	*τ*_3_ (ps)	*I*_3_ (%)
182 ± 3	42 ± 1	312 ± 2	52 ± 1	1342 ± 150	6 ± 0.2

Temperature dependent Doppler broadening of the annihilation radiation indicates the momentum distribution of annihilating electrons, which provides important fundamental insight into the temperature dependent thermoelectric properties of the nanocrystalline AgCuS during the (β–α) transition. The Doppler broadening spectra have been analyzed by evaluating the S-parameter defined by the ratio of the counts in the central area of the annihilation photo-peak and the total area of the photo-peak. In Fig. 9b, we present the temperature dependent S-parameter curve for nanocrystalline AgCuS (∼40 nm) obtained after 30 min of reaction. In the nanocrystalline AgCuS, the variation of the S-parameter with temperature is almost flat (within error limit) during the (β–α) transition and beyond 450 K the fall off of S-parameter is shallow. In the case of the bulk AgCuS,^13^ during the (β–α) transition, onset of the S-parameter starts at ∼360 K and it reaches a maximum at ∼385 K, then it falls rapidly compared to that of the nanocrystalline sample. The nanocrystalline AgCuS sample is enriched with Ag vacancy like defects and Ag vacancy clusters, but the rise of the S-parameter with temperature in the temperature interval of 273 K to 393 K is significantly absent. This result indicates that the Ag vacancy is less diffusive in the nanocrystalline AgCuS compared to that of its bulk counterpart at high temperatures. Movement of Ag/Cu atoms during the (β–α) transition occurs through the Ag vacancy. Thus migration of the Ag vacancy is essential for phase transformation. The large grain size in the bulk crystals results in long range periodicity in the crystal lattice, hence Ag vacancies are delocalized in nature, which essentially favors the p–n–p type conduction switching in bulk AgCuS.^13^ However, in the AgCuS nanocrystals, due to low periodicity of the crystal lattice (*i.e.* due to smaller size), the Ag vacancy is localized in nature which demands creation of more Ag vacancies during the (β–α) transition. This resulted in the leaching out of the Ag from the AgCuS nanocrystals followed by the segregation at the surface or grain boundaries. Ag leaching from the AgCuS nanocrystals injects more positively charged carriers, which makes the AgCuS nanocrystal p-type throughout the measured temperature range. Temperature dependent PXRD data also shows the appearance of the Ag peak along with the hexagonal (α) and cubic (δ) AgCuS phases on heating from orthorhombic (β) AgCuS nanocrystals (Fig. 1c).

Previous electronic structure calculations on the bulk AgCuS during the (β–α) transition indicated that the contribution from hybridized Cu–S orbitals to the overlapping valence and conduction band gave rise to the semimetallic character of the intermediate structure that was responsible for the p–n–p type conduction switching. Although the signature of Cu–S bond vibration was present in the Raman spectra of the bulk AgCuS during the (β–α) transition, the Raman spectra of nanocrystalline AgCuS does not show any Cu–S bond vibration during the (β–α) transition. This result also indicates the absence of a semimetallic intermediate electronic state during the (β–α) transition in nanocrystalline AgCuS.

## 4. Conclusions

In conclusion, nanocrystalline AgCuS was synthesized for the first time by a simple capping free solution phase reaction at room temperature. Temperature dependent synchrotron powder X-ray diffraction, heat capacity (*C*_p_) and Raman spectroscopy measurements clearly indicate that the nanocrystalline sample undergoes two distinct order–disorder phase transitions at high temperatures. A superionic order–disorder phase transition tailors the thermoelectric properties in AgCuS nanocrystals and the fundamental understanding was developed based on temperature dependent Raman spectroscopy, positron annihilation spectroscopy and optical absorption experiments. Size reduction to the nano-dimension resulted in the vanishing of p–n–p conduction switching in AgCuS due to the following combined reasons: (a) higher band gap of AgCuS nanocrystals than that of the bulk counterpart, which is not appropriate for the creation of a semimetallic electronic state and the shifting of the Fermi level in the conduction band during the first order–disorder phase transition, (b) absence of hybridized Cu–S states during the (β–α) transition, which were otherwise responsible for the creation of an intermediate semimetallic state, (c) high surface to volume ratio in nanocrystalline AgCuS, which gives rise to the formation of more Ag vacancies and increases the p-type carrier concentration and (d) Ag leaching from AgCuS nanocrystals at elevated temperature injects more positively charged carriers into the system, which makes the AgCuS nanocrystal p-type throughout the measured temperature range. Our findings demonstrated that the p–n–p type conduction switching and thermoelectric properties of the bulk noble metal based chalcogenides can be tuned by reducing the crystallite size to nanometer scale. The discovery of an order–disorder phase transition in nanoscale materials and its effect on the electronic structure can bring novel phenomena with unusual properties.

## Supplementary Material

Supplementary information
