# Supplementary material for: The effect of order–disorder phase transitions and band gap evolution on the thermoelectric properties of AgCuS nanocrystals
Source: Chem Sci. 2015 Oct 8;7(1):534–43. doi: 10.1039/c5sc02966j (PMC5952868; doi:10.1039/c5sc02966j)
Supplement: Supplementary file 1 [file SC-007-C5SC02966J-s001.pdf]

Electronic Supplementary Material (ESI) for Chemical Science.
This journal is © The Royal Society of Chemistry 2015

Electronic Supplementary Information (ESI)

Effect of Order-Disorder Phase Transition and Band Gap Evolution on the Thermoelectric Properties of AgCuS Nanocrystals†

Satya N. Guin,[a] Dirtha Sanyal[b] and Kanishka Biswas*[a]

[a]*New Chemistry Unit, Jawaharlal Nehru Centre for Advanced Scientific Research (JNCASR), Jakkur P.O., Bangalore 560064, India*

[b]*Variable Energy Cyclotron Centre, 1/AF Bidhannagar, Kolkata 700064, India*

** Email: kanishka@jncasr.ac.in*

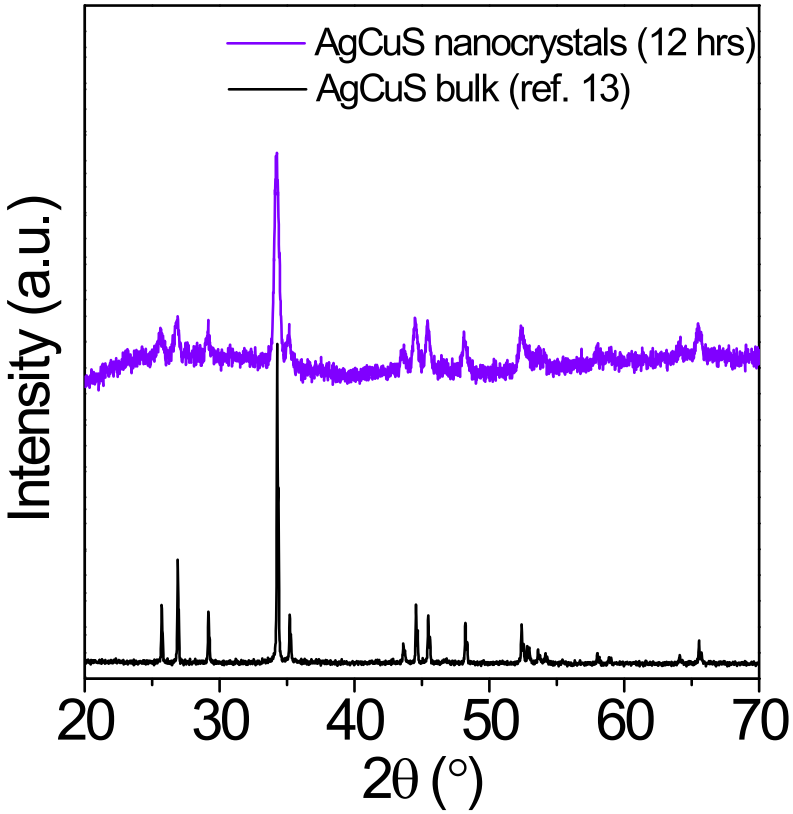

Fig. S1 Powder XRD pattern for AgCuS nanocrystals obtained after 12 hrs of reaction time measured at lab source (Cu K_{α}; $\lambda = 1.5406$ Å) with PXRD of bulk AgCuS.[13]

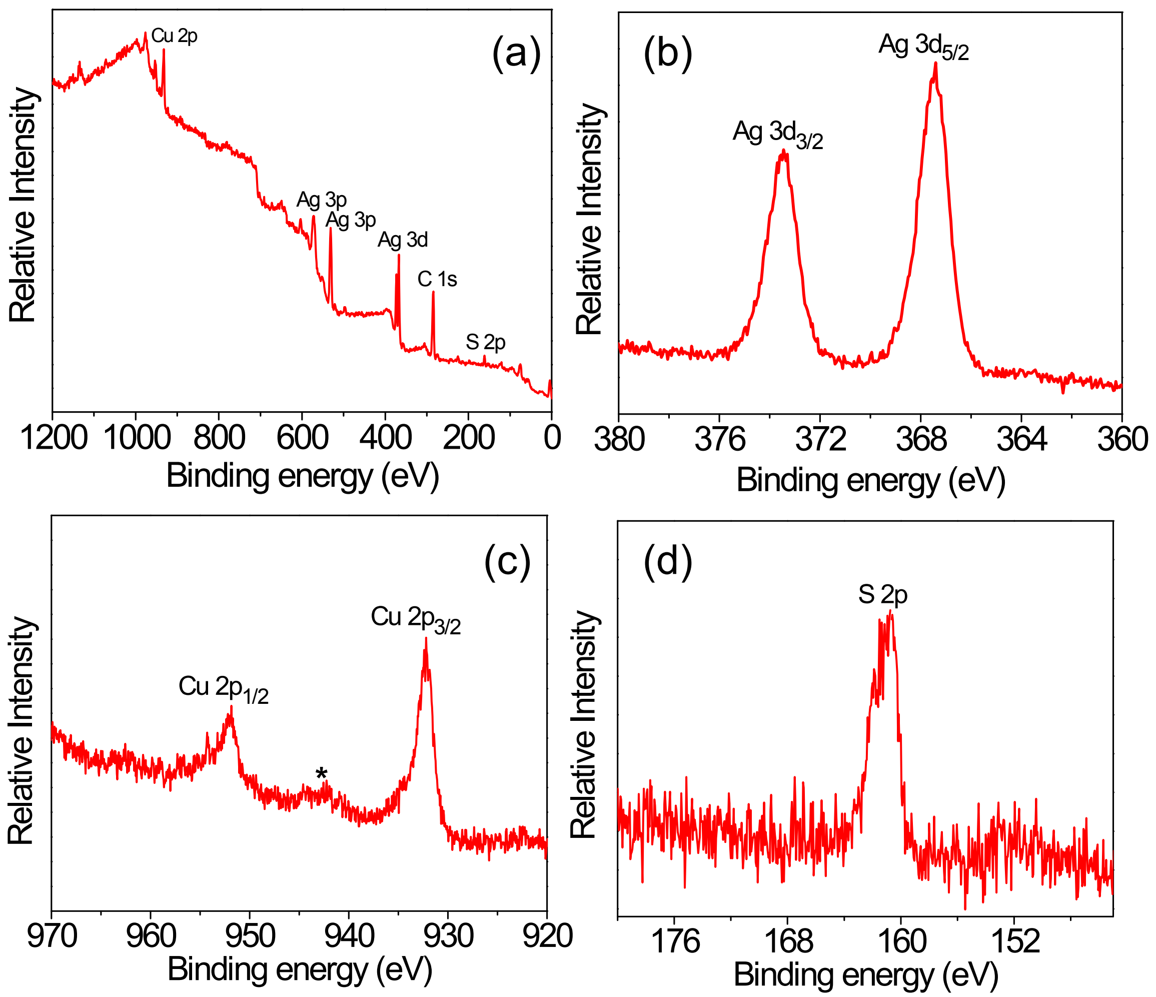

Fig. S2 XPS spectra of AgCuS nanocrystals (30 min reaction) (a) XPS survey scan (b) Ag 3d, (c) Cu 2p and (d) S 2p spectra. An additional peak (* marked) in Cu 2p was due to presence of small amount of Cu(II).

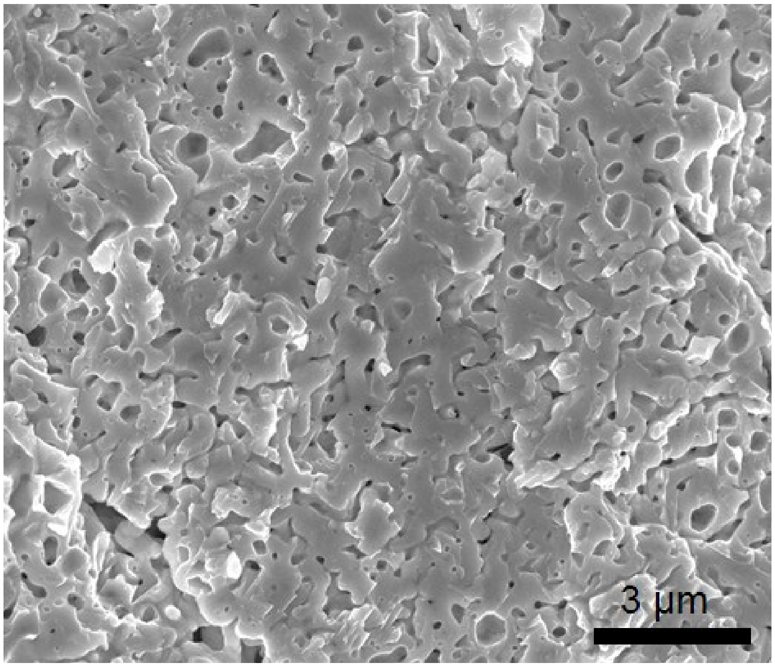

Fig. S3 FESEM image after sintering of AgCuS nanocrystals at 573 K for 3 hrs.

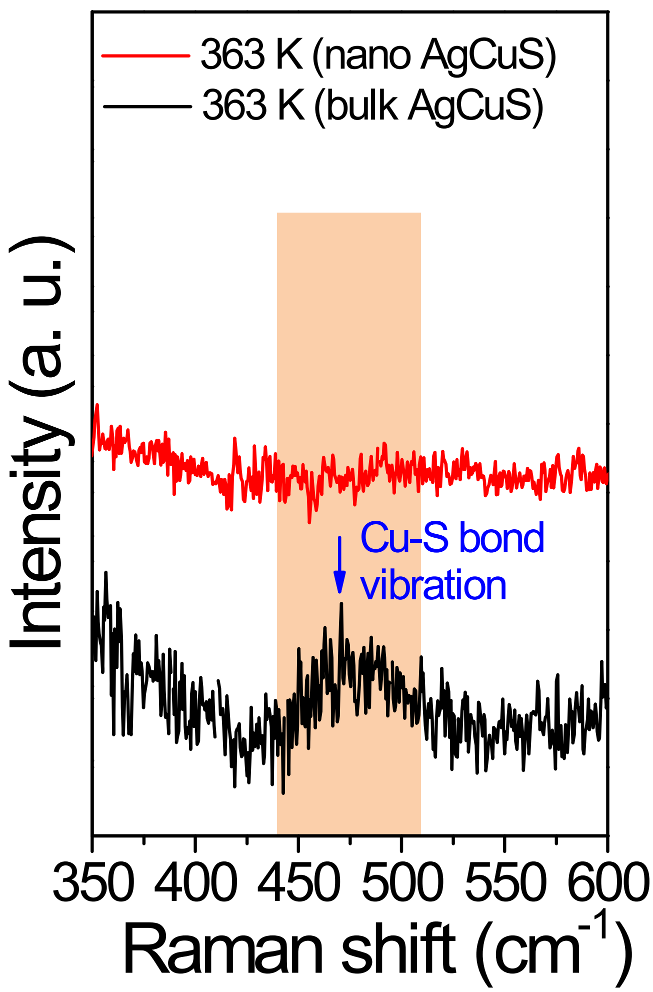

Fig. S4 Raman spectra of bulk and nanocrystalline AgCuS (30 min reaction), in the range of 350-600 cm^{-1}, indicating the absence of Cu-S bond vibration in nanocrystalline sample during orthorhombic (β) to hexagonal phase (α) transition.

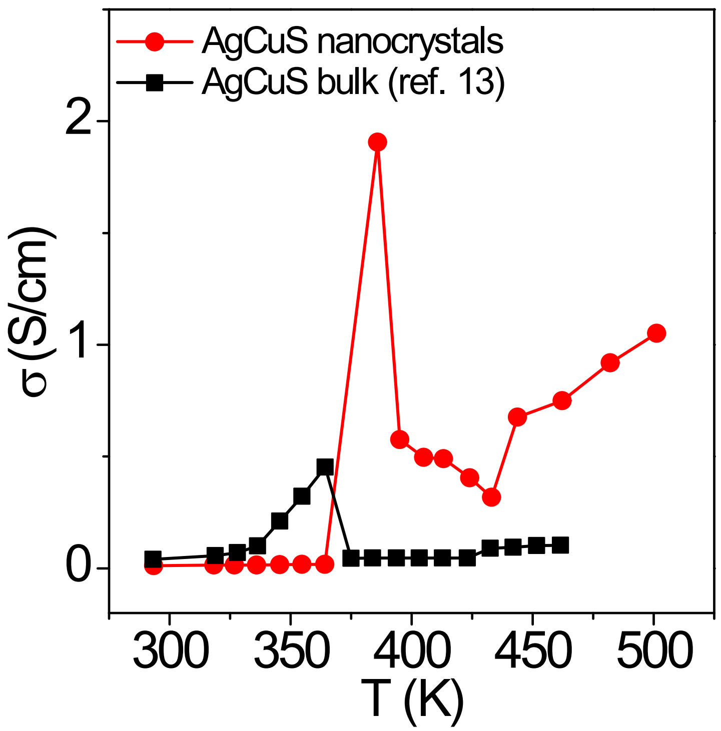

Fig. S5 Temperature dependent electrical conductivity (σ) of nanocrystalline AgCuS compared with its bulk counterpart[13].

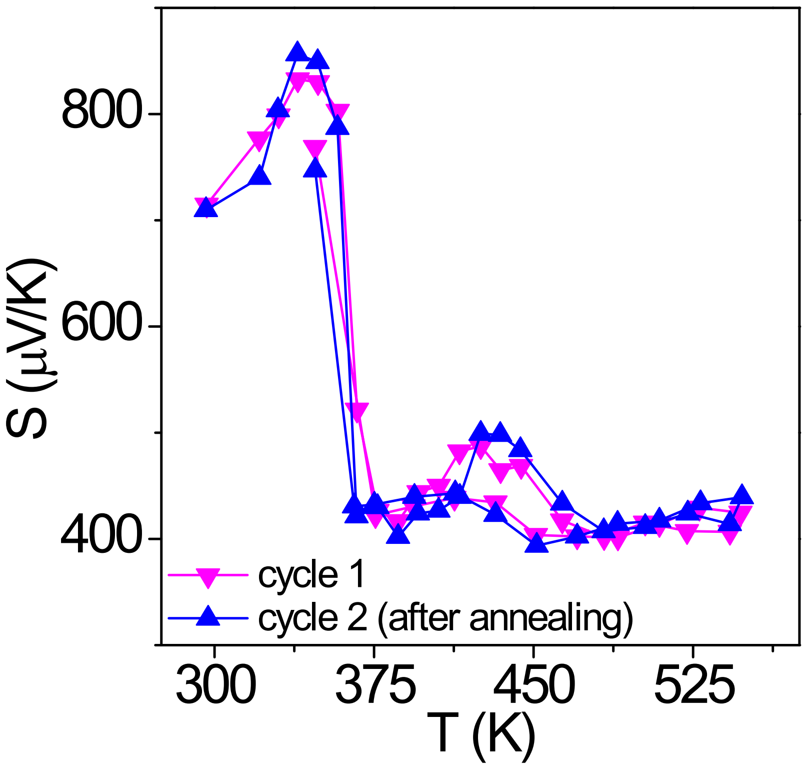

Fig. S6 Temperature dependent two cycle heating cooling Seebeck coefficient data for nanocrystalline AgCuS (30 min reaction).

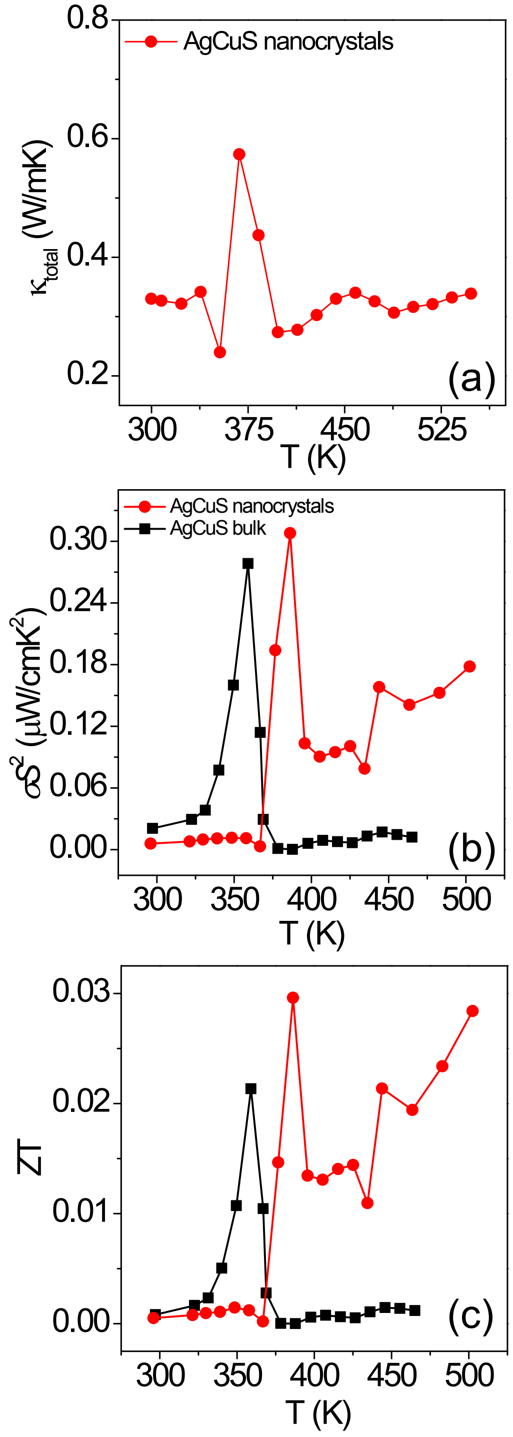

Fig. S7 Temperature dependent (a) thermal conductivity (κ_{total}) of AgCuS nanocrystals obtained after 30 min of reaction. (b) and (c) are comparative temperature dependent power factor (σS^2) and thermoelectric figure of merit (*ZT*) of bulk and nanocrystalline AgCuS, respectively.

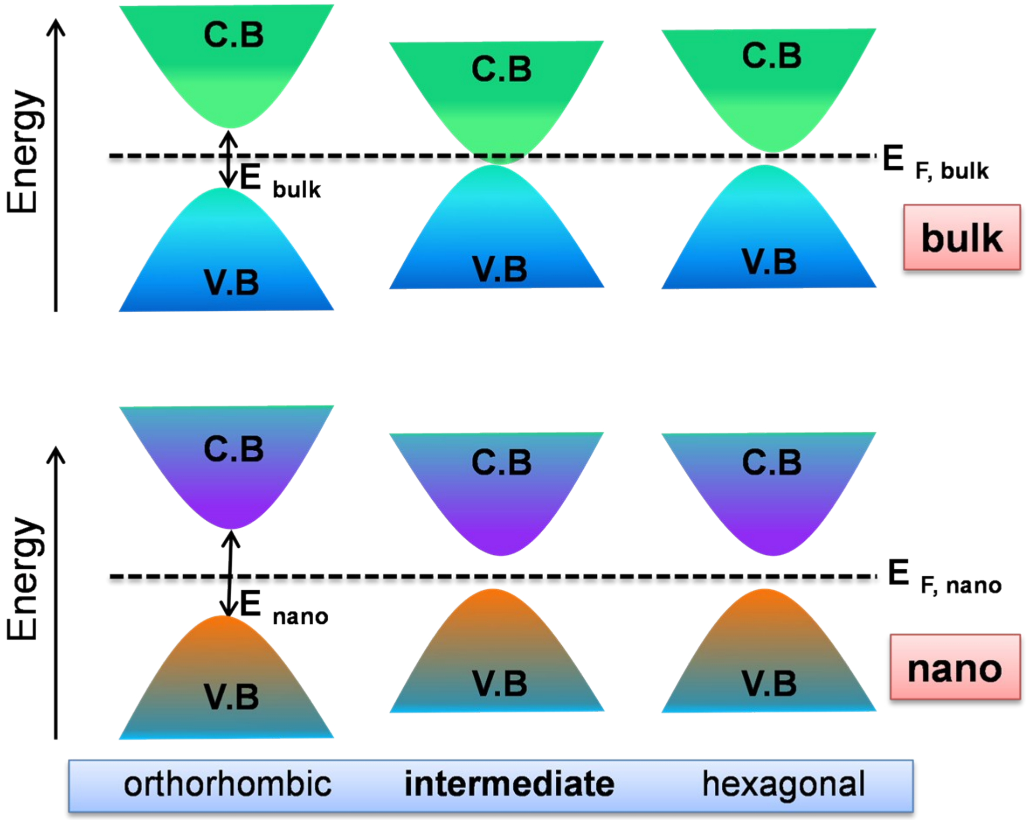

Fig. S8 Schematic representation of changes of the band gap of bulk and nanocrystalline AgCuS during orthorhombic (β) to hexagonal (α) phase transition.
